# Antioxidant Activity of Different Phenolic‐Rich Fractions Obtained From Peels of Turkish Dark Purple Eggplant

**DOI:** 10.1002/fsn3.70521

**Published:** 2025-06-27

**Authors:** Assia Djouadi, Hakan Yılmazer, Anfal Djouadi, Bilal Çakır

**Affiliations:** ^1^ Department of Metallurgical and Materials Engineering, Faculty of Chemical and Metallurgical Yıldız Technical University Istanbul Turkey; ^2^ Health Biotechnology Joint Research and Application Center of Excellence Esenler Istanbul Turkey; ^3^ Department of Cellular and Molecular Biology, Faculty of Natural Sciences and Life University of El‐Oued El‐Oued Algeria; ^4^ Halal Food R&D Center İstanbul Sabahattin Zaim University İstanbul Turkey

**Keywords:** DPPH antioxidant activity, eggplant skin, extraction, HPLC, polyphenols, spectrophotometry

## Abstract

This study investigated the extraction of phenolic compounds and the antioxidant potential of eggplant peel using various extraction methods and GRAS solvent systems. Ethanol absolute and (EtOH: water) (70:30) system solvents were used separately to extract phenolic compounds, while the (EtOH: water: citric acid) (70:30:0.5) system was utilized for anthocyanin extraction. Otherwise, to test the antioxidant activity of anthocyanin separately, an aqueous anthocyanin‐rich fraction was recovered from eggplant peel crude extract using successive liquid/liquid extractions with solvents of different polarities. The presence of total phenolics, anthocyanins and proanthocyanidin were detected, and HPLC analysis was realized. The antioxidant activity was performed by the (DPPH) free radicals method. Soxhlet extraction using absolute ethanol provides a high yield of phenolic compounds. Otherwise, the anthocyanin extract showed a high level of anthocyanin contents. The liquid chromatographic analysis presented an important percentage of flavonoids. Soxhlet extract was the most effective in scavenging free radicals.

## Graphical abstract

Polyphenol contents of Turkish eggplant peels were extracted by different solvent systems and extraction methods. Liquid–liquid extraction was performed for anthocyanin separation. Qualitative and quantitative differences in phenolic profiles were observed. Soxhlet extraction by absolute ethanol showed the highest total phenolic content and strongest antioxidant activity. Hot water bath extraction with an acidic medium yielded a high extraction of flavonoid compounds.

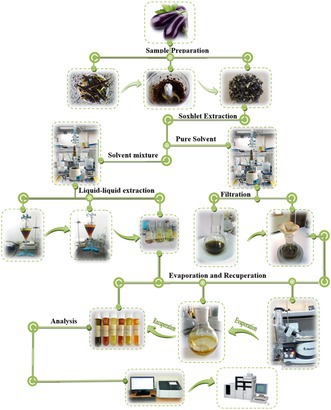

## 1 Introduction

For a healthy lifestyle, it is commonly recommended to have a diet that is abundant in fresh fruits and vegetables. These foods are important sources of both nutrients and non‐nutrients (O'Shea et al. 2012), such as antioxidants, phytosterols, and fiber (Martins et al. 2013; Massias et al. 2015; Morales‐Soto et al. 2014). Apart from providing essential nutrients, plant‐based foods also contain biologically active components known as phytochemicals, including polyphenols, terpenoids, alkaloids, phytosterols, and organosulfur compounds, as well as dietary fiber. Bioactive compounds are generally defined as substances that have the biological potential to positively impact health (Lee and Kim 2022). On the other hand, as per the Food and Agriculture Organization's findings, approximately one‐third of the edible portions of food intended for human consumption is lost or wasted on a global scale. This corresponds to an estimated 1.3 billion tons per year, encompassing not just food processing wastes but also encompassing what is referred to as “food losses” (Galanakis 2012). The process of converting raw materials into food products results in the generation of “waste,” which includes the edible portion of food that is lost, discarded, or degraded throughout various stages of the food supply chain (Massias et al. 2015). Fruits and vegetables contain a wealth of bioactive compounds, including phenolic compounds, carotenoids, and vitamins, which are present throughout their entire tissue. Interestingly, the by‐products that are often wasted can contain similar or even higher levels of antioxidant and antimicrobial compounds compared to the final food products (Gowe 2015). Scientific investigations have demonstrated that phytochemicals and essential nutrients are predominantly found in the peels, seeds, fruits, and vegetables (Hussain et al. 2022). Given the growing prevalence of infectious and chronic diseases, there is an urgent need to discover natural agents with novel mechanisms of action. The vast availability of chemical diversity in natural products offers limitless opportunities for the development of new drug leads (Gowe 2015). Fruit and vegetable peels are discarded as waste into the environment by households and food‐processing industries. Nevertheless, these sources are currently underutilized due to a limited understanding of their nutritional and economic significance (Gowe 2015; Pathak et al. 2016).

As per the findings of references (Galanakis 2012; Hussain et al. 2022; Kumar et al. 2020), it has been documented that fruit and vegetable wastes contain significant quantities of secondary metabolites. These waste materials have been the subject of research regarding the extraction of phenolic molecules, dietary fibers, and other biologically active metabolites.

Over the past decade, there has been an increasing focus among researchers and food manufacturers on polyphenols. These compounds are recognized as potent phenolic antioxidants, and to date, more than 8000 different structures have been identified. Polyphenols are abundant micronutrients found in our diet, and they are particularly concentrated in a wide range of fruits and vegetables. Their significance has attracted considerable attention in recent years due to their essential contributions to maintaining good health. They play a vital role in regulating metabolism, weight, and chronic diseases, support cell proliferation, and help reduce the risk of coronary heart diseases, neurodegenerative diseases, and certain forms of cancer (Cory et al. 2018; Manach et al. 2004; Colak et al. 2022).

The primary factor driving this interest stems from the acknowledgement of polyphenols' antioxidant properties, their abundant occurrence in our diet, and their potential role in preventing various diseases linked to oxidative stress, including cancer, cardiovascular diseases, and neurodegenerative disorders (Manach et al. 2004).

Polyphenols, plant compounds with a wide distribution in nature, have garnered attention with the identification of around 8000 different structures (Colak et al. 2022). These molecules serve as secondary metabolites in plants and are primarily involved in defense mechanisms against ultraviolet radiation and attacks by pathogens. Within higher plants, numerous molecules characterized by a polyphenol structure have been identified, totaling several thousand compounds. This structure is defined by the presence of multiple hydroxyl groups attached to aromatic rings. Additionally, edible plants have been found to contain several hundred distinct polyphenolic compounds (Manach et al. 2004). Despite their vast diversity, all these polyphenols share a fundamental structural feature: an aromatic ring that possesses at least one hydroxyl substituent, commonly known as a phenol (Stalikas 2007).

Phenolic compounds within the plant kingdom can be categorized into various groups based on the number of phenol subunits they possess and the structural elements that connect these subunits to one another. This broad categorization of “plant phenolics” includes various types such as simple phenols, phenolic acids, coumarins, flavonoids, stilbenes, hydrolysable and condensed tannins, lignans, and lignins (Manach et al. 2004; Stalikas 2007).

Each matrix has a distinct composition, and phenolics have variable solubility characteristics because of their structural differences (Condurache et al. 2021). Moreover, the non‐uniform distribution of phenolic compounds within the same plant contributes to the variability in their stability. While certain phenolic compounds exhibit stability, others are susceptible to oxidation, thermolability, or volatility. As a result, some parts of the plant may contain higher concentrations of stable phenolic compounds, while other parts may have lower concentrations or may contain phenolic compounds that are more prone to degradation or chemical changes. The extraction of polyphenols from their sources can be a challenging task due to the high levels of enzyme activity present in most foods and plants. Therefore, careful consideration must be given to the selection of the extraction process to prevent any chemical changes to the target compounds (Alara et al. 2021; Robards 2003). At present, there is no universally accepted method for effectively extracting all phenolic compounds or a particular group of them from plant materials. Hence, the primary consideration in selecting an extraction process revolves around the type of sample and the targeted compounds, which may include total phenolics, specific classes of phenolics, or others (Alara et al. 2021). Therefore, to recover phenolic compounds from eggplant peels, it is necessary to explore various extraction methods and evaluate the efficiency of different solvent types. The study utilized three solvents: absolute ethanol, aqueous ethanol, and aqueous ethanol acidified with 0.5% citric acid. Ethanol was specifically chosen for its ability to achieve a high recovery rate of phenolic compounds, its nontoxicity, and its suitability for polar compounds (Condurache et al. 2021; Hasbay and Galanakis 2018).

The use of Soxhlet extraction is recognized as a straightforward method for isolating compounds from bark. This methodology offers several advantages. Firstly, it is a simple technique that does not necessitate any specialized equipment. Secondly, it ensures high reproducibility. Thirdly, it allows for successive extraction runs using solvents of different polarities and in various polarity sequences. Lastly, the extract can be easily obtained by distilling the solvent, enabling solvent recovery, which promotes both the economic and environmental sustainability of the overall process (Autor et al. 2022).

Extraction serves as a key step in the retrieval and isolation of bioactive phytochemicals from plant materials prior to analysis. Its effectiveness is influenced by factors such as the chemical properties of the compounds, the extraction method utilized, the particle size of the sample, and the presence of any interfering substances. Otherwise, efficient extraction methods are essential for various applications involving polyphenols and other biomolecules derived from biomass. Consequently, a range of extraction systems and techniques have been employed to address this need (Celli et al. 2015; Elez Garofulić et al. 2013; Ghafoor et al. 2011; Marqués et al. 2013; Paes et al. 2014). In addition, if the objective is to remove unwanted phenolics and non‐phenolic substances like waxes, fats, terpenes, and chlorophylls, additional steps may be required. The selection of an appropriate extraction method depends on the specific type of phenolic compound to be extracted. Among various extraction methods, solvent extraction is the most commonly used approach, with certain solvents being preferred for specific phenolic compounds. In many cases where the extracted phytochemical is intended for use as a nutraceutical or clinical standard, ethanol and aqueous ethanol solutions are commonly selected as solvents (Routray and Orsat 2013). However, Soxhlet extraction stands out as the predominant technique for phenolic compound extraction due to its cost‐effectiveness, simple operation, suitability for both initial and bulk extraction, efficient recovery of extracts, shorter extraction time, and lower solvent consumption compared to conventional methods like maceration or percolation (Seidel 2012). Moreover, Soxhlet extraction has long been recognized as a reliable technique with superior performance, outperforming other conventional extraction techniques, except when dealing with thermolabile compounds (Luque de Castro and García‐Ayuso 1998). Furthermore, in comparison to alternative conventional methods, Soxhlet extraction allows for higher yields using a smaller quantity of solvent (Alara et al. 2018).

In this context, the main goal of this study is to highlight the potential of eggplant peels as a valuable source of bioactive compounds for functional food development. More so, it can provide important information to be valorized and applied as a healthy product in the nutritional fabrication and medical field, while also presenting an opportunity to mitigate peel waste accumulation in the environment.

Thus, considering the nutritional and bioactive potential of *Solanum melongena L*. fruit peel, this work addresses the utilization of different extraction methods and solvents to efficiently recover phenolic compounds in order to identify the optimal extraction methodology and obtain a polyphenol‐rich extract from Turkish eggplant peel. It also aims to contribute to the chemical characterization of this fruit and evaluate its bioactive properties.

## 2 Materials and Methods

### 2.1 Chemicals and Reagents

2,2‐Diphenyl‐1‐picrylhydrazyl (DPPH), Folin–Ciocalteu reagent, Gallic acid, catechin, vanillin, quercetin, rutin, delphinidin‐3‐O‐rutinoside, and sodium carbonate were obtained from Sigma/Aldrich (St. Louis, MO, USA). All other chemicals and reagents were of analytical grade.

### 2.2 Plant Materials

The variety of *S. melongena* (eggplant) with the local name of Patlıcan was purchased fresh in October 2022 from a local market in Güngören, Turkey.

### 2.3 Preparation of Samples for Extraction

Bright dark purple color eggplants, free from wilts, discolored spots, and white fungal patches were selected for our study. The fruits were washed carefully, and the peels were removed with a knife in strips of uniform thickness. Post‐peeling, reducing the sample size of peels by grinding was performed for extraction. The fresh peels were homogenized with a blender. Fresh samples were kept at 4°C until analyzed.

### 2.4 Extracts Preparation

#### 2.4.1 Polyphenol Soxhlet Extraction

The fresh peel samples were Soxhlet extracted with absolute ethanol at a rate of 1:10 (raw material: solvent) (Oluwaseun R. Alara et al. 2018), at 60°C for 2 h under constant stirring. The samples were then filtered using Whatman filter N^o^ 4 paper; the filtrate was recovered and evaporated to dryness at a reduced temperature. After drying, the residue was weighed and re‐dissolved in ethanol and filtered. The ethanolic extract was stored in an airtight glass bottle in a refrigerator at 4°C for further analysis.

#### 2.4.2 Hot Water Bath Extraction

For polyphenols extraction, a second 50 g of the fresh sample was treated with 200 mL of 70% (v/v) ethanol solution. Additionally, another two fresh samples (50 g) were extracted separately using the same volume (200 mL) of ethanol: water (70:30 v/v); but acidified with 0.5% citric acid for recovering anthocyanins. The extractions were performed at 50°C for 30 min under constant stirring using a water bath. At the end of each extraction, the extracts were subjected to filtration with the Whatman paper. Then, the all filtrates were recovered and further portioned in a separating funnel using petroleum ether to remove lipophilic compounds. According to the fourth extract of anthocyanin, liquid/liquid extraction methods were performed using solvents of increasing polarity (petroleum ether, ethyl ether and ethyl acetate), yielding four fractions (petroleum ether, ethyl ether, ethyl acetate and a mixture of EtOH: water: citric acid).

The peel extract was first extracted with petroleum ether (40°C–60°C) (fraction I), ethyl ether (fraction II) and ethyl acetate (fraction III) in succession (each of the steps was repeated three times to ensure complete extraction in each case), obtaining an aqueous residue (containing all the anthocyanin compounds in the sample) and organic phases; fraction I is rich in fatty substances, fraction II contains free flavonoids, whereas the ethyl acetate fraction contains flavonols, phenolic acids, and catechins. After drying in a vacuum using a rotary evaporator, all dry fractions were sealed in a glass bottle and stored at 4°C until used.

### 2.5 Determination of Total Phenolic and Flavonoid Content

#### 2.5.1 Total Phenolic Content

The total phenolic content was tested using a colorimetric Folin–Ciocalteu method (Scalbert et al. 1989). Appropriately diluted test extracts (150 μL) were mixed with 750 μL of Folin–Ciocalteu reagent followed by the addition of 3 mL of 7.5% aqueous sodium carbonate (Na_2_CO_3_). Then, the above mix was vortexed and incubated in the dark at room temperature for 60 min. The absorbance of the mixture was read at 735 nm against a reagent blank using a UV–Visible spectrophotometer. Quantification was done with respect to the standard of gallic acid. A standard curve was prepared using different concentrations of gallic acid solutions to quantify the phenolic compounds. The total phenolic content was expressed as gallic acid equivalents (GAE) in mg per g dry extract. All samples were analyzed in triplicate.

#### 2.5.2 Total Condensed Tannin Content (CTC) Assay

The analysis of condensed tannins (Proanthocyanidins) was carried out according to the method of Sun et al. (1998). A mixture of 3 mL of 4% ethanolic vanillin solution (4%, v/v) and 1.5 mL of concentrated HCl were added to 0.5 mL of suitably diluted sample and then vortexed. The red mixture was allowed to stand for 15 min and the absorbance was measured at 568 nm using a UV–Visible spectrophotometer. The amount of total proanthocyanidin was expressed as mg (+)‐catechin equivalents/g of dry extract (mg CE/g) using the equation obtained from the calibration curve. All samples were analyzed in three replications.

#### 2.5.3 Total Anthocyanins Content

The total anthocyanins content was quantified using the pH‐differential method (Cao 2023), and the results were expressed in milligrams of delphinidin 3‐O‐rutinoside per gram of dry weight of peel extract (mg D3G/g dw) ± SD. The pH differential method is based on the color change of monomeric anthocyanins at two different pH values (1.0 and 4.5) (Taghavi et al. 2022). Briefly, each sample was dissolved in 0.025 M potassium chloride (KCl) buffer solution at pH = 1.0 and 0.4 M sodium acetate (CH_3_COONa) buffer solution at pH = 4.5. The absorbance was determined at 520 nm in a UV–Visible spectrophotometer. The dilution factor for each sample was firstly determined by dissolving the sample in KCl buffer pH 1.0 until its absorbance at 520 nm obtained less than 1.2 versus the KCl buffer pH 1.0 as a blank. The sample was then dissolved in KCl buffer pH 1.0 (allowed to stand for another 15 min) and CH_3_COONa.3H_2_O buffer pH 4.5 (allowed to stand for another 5 min) based on the dilution factor.

The color changes were measured using a spectrophotometer at *λ* = 520 nm (for the delphinidin 3‐O‐rutinoside) and *λ* = 700 nm (for correction factor) at pH 1.0 (potassium chloride buffer) and pH 4.5 (sodium acetate buffer). The final absorbance (A) was calculated using Equation (1).

(1) $$A={\left(A_{520}-A_{700}\right)}_{\mathrm{pH}.01}-{\left(A_{520}-A_{700}\right)}_{\mathrm{pH}.4.5\;}$$

The TAC was assessed according to the equation obtained from the calibration curve of delphinidin 3‐O‐rutinoside standard solution. All samples were analyzed in three replications.

### 2.6 Antioxidant Activity

#### 2.6.1 DPPH Radical Scavenging Activity

The antiradical activity of each peel eggplant extract was determined using a stable 2,2‐diphenyl‐1‐picrylhydrazyl radical (DPPH^•^) assay (Brand‐Williams et al. 1995). In its radical form, DPPH^•^ has an intense violet color with a maximum absorbance band at 517 nm, which disappears and turns colorless as unpaired electrons are scavenged by antioxidant compounds. An 800 μL daily prepared DPPH^•^ ethanolic solution (0.004%) was mixed with 200 μL of various concentrations of each test extract. After shaking, the mixture was allowed to react in the dark at room temperature for 30 min. The absorbance was measured spectrophotometrically at 517 nm using a UV–visible spectrophotometer. A tube containing ethanol and DPPH solution was used as a control, whereas ethanol alone was used as a blank. The antiradical activity was expressed as 50% inhibition (IC_50_) (mg/mL), the concentration of extract required to scavenge free radicals by 50%. A 50% inhibition was estimated from a calibration curve analyzed by linear regression, plotting percent radical scavenging activity against test extract concentration. Radical‐scavenging activity (RSA%) was calculated as follows:

$$\mathrm{RSA}\%=\left(1-\frac{A_{1}}{A_{0}}\right)\times 100$$

where *A*_0_ is the absorbance of the control reaction and *A*_1_ is the absorbance of the test extract.

### 2.7 Antioxidant Identification

#### 2.7.1 HPLC Analysis of Polyphenols

The HPLC analysis of the phenolic compounds was conducted using an Advanced i‐Series UPLC system (SHIMADZU Inc., Kyoto, Japan), equipped with a DAD. The separation was conducted using a Waters Sunfire C18 reverse‐phase chromatography column, 250 mm length, 4.6 mm width, and particle size 5 μm. The phenolic standard solutions and mixtures were injected into the system using an autoinjector. Different isocratic and gradient mobile phases were tested at different flow rates and column temperatures to find a suitable separation method for the standards.

In the isocratic method chosen after a series of preliminary studies, a mixture of methanol and ultrapure water (1:1) is used. The total run time of the method was 30 min. A constant flow rate of 1.00 mL/min and column oven temperature of 40°C were used. Following the analysis of UV–Vis spectra of individual phenolic standards, two wavelengths (260 and 280 nm) were selected for analysis in this research using HPLC‐DAD. The results were expressed in mg of each phenolic compound/g of extract.

### 2.8 Statistical Analysis

All results were reported as means ± standard deviation (SD) of all samples. A one‐way analysis of variance (ANOVA) procedure and Tukey test at *p* ≤ 0.05 were applied to test differences between means using the Minitab software, Version 21 (SAS Institute Inc., Cory, NJ).

## 3 Results and Discussion

In this study, two different common extraction methods and different solvent systems were tested, in order to select the most suitable extraction, which can lead to getting an extract rich in important content of polyphenols with high antioxidant properties. In fact, Soxhlet and water bath extraction methods were selected in this study (Autor et al. 2022).

### 3.1 Total Polyphenol Contents

The results examination showed significant differences in eggplant peel total polyphenols when comparing the four extracts (Table 1). The extracts with the total phenolic content values, in decreasing order, were Soxhlet extract (SPE) > crude anthocyanin extract (CAE) > separated anthocyanin extract (AF) > crude water bath extract (WPE), with total TPC values of 856.71, 28.97, 24.30, and 22.75 mg GAE/g DR, respectively.

**TABLE 1:** Content of total phenols, condensed tannins, and anthocyanins of different extracts obtained.

Content	SPE	CAE	AF	WPE
	mg/g dry extract (mean ± SD)			
Total polyphenols (GAE)	856.71 ± 16.06^a^	28.97 ± 1.42^b^	24.30 ± 4.75^b^	22.75 ± 2.88^b^
Total anthocyanins (D‐O‐R)	nd	157.29 ± 2.43^a^	112.38 ± 4.75^b^	29.21 ± 0.45^c^
Condensed tannis (CE)	nd	368.66 ± 2.96^a^	346.49 ± 1.48^b^	131.36 ± 1.54^c^
IC_50_%	0.046 ± 0.002^d^	0.523 ± 0.001^c^	0.780 ± 0.001^a^	0.613 ± 0.001^b^

*Note:* Data represent the mean ± standard deviation of triplicate determinations (*n* = 3). ^a–d^Means in a row with different letters indicate a statistically significant difference (*p* < 0.05); nd: not detected.

In fact, the Soxhlet extract had significantly (*p* < 0.05) the highest phenolic content (856.709 mg GAE/g DR) whereas, the water bath extracts presented the lowest content (less than 30 mg GAE/g DR). This important variability pointed out the solvent and method influence on the extractability of antioxidant compounds, in particular on the extractability of phenolics. These data agree with the previous study, which found that the Soxhlet method exhibits the highest content of phenolic compounds (Bat 2021).

Moreover, according to statistical results reported in the literature (Deng et al. 2013; Fu et al. 2011) about the total phenolic contents of 62 and 56 commonly consumed fruits and vegetables, respectively, the total phenolic contents of the lipophilic fraction were much higher than those of the hydrophilic fraction. Thus, the pretreatment with petroleum ether to remove chlorophyll, carotenoids, and sterols could contribute to separating also lipophilic phenols from extracts, which explains the lowest values of phenolic contents in different shaking water bath extracts. In respect to another study, which examined different Turkish eggplant cultivars based on their shape: Topan (wider than long), Uzun (longer), Beyli (longer than wide), and Domates (tomato‐shaped), it was found that the total phenolic content varied from 615 in MM738 to 1389 mg/kg in Eskisehir Tombul, and the mean phenolic content was 992 ± 46 (SE) mg/kg for all of the cultivars (Okmen et al. 2009; Sharma et al. 2021).

The phenolic content profile of dark purple eggplant, as investigated in various studies (Pantuzza Silva et al. 2020; Singh et al. 2017), revealed that the extracted compounds from eggplant peels possess a higher quantity of individual phenolic compounds compared to those found in the eggplant pulp. Additionally, the analyzed extracts from both the eggplant peels and pulp contain greater quantities of most of the phenolic compounds. Furthermore, the literature (Pantuzza Silva et al. 2020; Singh et al. 2017) demonstrates that the extracts obtained from the eggplant peel consisted of various classes of compounds, such as phenolic acids, anthocyanins, and flavonols.

### 3.2 Total Anthocyanin and Condensed Tannin Contents

The results exhibited significant differences in total anthocyanin and condensed tannin content according to the solvent power and extracting method used (Table 1). In fact, total anthocyanins content was remarkably superior in the crude anthocyanin extract (157.29 mg D‐O‐RE/g DR) as compared to other extracts examined (112.38 and 29.21 mg CE/g DR, respectively). Furthermore, the AF fraction presents content of anthocyanins significantly less than the one in CAE extract, which confirms that some anthocyanins compounds from the corresponding extract are separated in ethyl acetate and/or diethyl ether during the liquid–liquid extraction step. That way the amount of these compounds decreased significantly in the extracts. Indeed, these results are consistent with earlier studies that have shown that flavonoids are highly soluble in ethyl acetate (Luo et al. 2002). Additionally, previous studies reported that ethyl acetate fraction obtained from the medicinal plant leaves showed the highest levels of flavonoids, in comparison with aqueous and acetonic fractions (Mariem et al. 2014; Pedreschi and Cisneros‐Zevallos 2007; Trabelsi et al. 2012). Otherwise, the lowest content of anthocyanins and condensed tannin compounds in the WPE sample approves the importance of an acidic medium for extracting flavonoids (Elzaawely and Tawata 2012; Niño‐Medina et al. 2017). In the same context, the data concerning condensed tannin contents are similar to those of total anthocyanin compounds, where the CAE sample was statistically the richest one (368.66 mg CE/g DR), which is in agreement with previous studies (Prior et al. 2005; Todaro et al. 2009), while the WPE samples showed the lowest significant values (131.36 mg CE/g DR). Furthermore, we observe the absence of anthocyanin and condensed tannin contents in Soxhlet extract. In the literature, condensed tannins can be degraded and oxidized to produce anthocyanins by heating in an acidic alcohol solution medium (Naumann et al. 2017; Qin et al. 2021). According to an earlier study, Additionally, high temperatures can improve extraction efficiency by increasing the solubility, diffusion coefficients, and mass transfer rate of the compounds. However, high temperatures combined with a longer extraction time can lead to phenolic degradation (Dranca and Oroian 2016; Hasbay and Galanakis 2018), which affirms that the extraction using the Soxhlet process at 60° for a long time led to the degradation of anthocyanin compounds.

**FIGURE 1:**
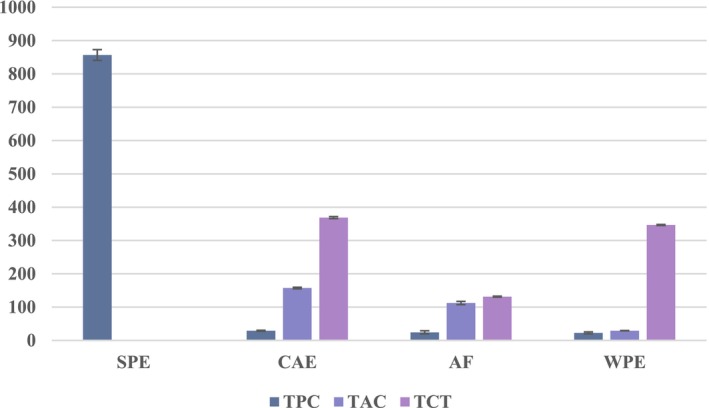
Content of total phenols, condensed tannins and anthocyanins in different examined extracts.

Likewise, the significant variability between the various extracts, in the phenolic compound contents, may be attributed to the extracting power of the solvent used and its chemical nature (organic or aqueous), structure, degree of polymerization and the interaction of these compounds with each other (Figure 1). Our results concerning the different classes of phenolic compound contents approve that obtained by Colak et al., who indicate that the black and purple eggplant are the cultivars with greater potential benefits in terms of their high phenolic contents and antioxidant properties than the white eggplant (Colak et al. 2022).

### 3.3 Antioxidant Capacities

Free radical scavenging is a fundamental antioxidant mechanism. Polyphenols have the ability to donate hydrogen, which can form a reduced state when interacting with free radicals. DPPH radical scavenging activity of gallic acid, delphinidin‐3‐O‐rutinoside, and all samples is shown in Table 2. It could be easily observed that the gallic acid exhibited a notable DPPH radical scavenging activity which ranged from 22.92% to 87.44% when the concentrations varied from 0.003 to 0.021 mg/mL. Initially, there was a positive linear correlation between the DPPH radical scavenging rate and the sample concentration. However, as the concentration continued to increase, the trend started to slow down, eventually resulting in an overall parabolic trend. A lower IC_50_ value corresponds to a higher antioxidant activity in the sample. The IC_50_ values of gallic acid and delphinidin‐3‐O‐rutinoside standards were 0.01 and 0.019 mg/mL, respectively.

**TABLE 2:** DPPH‐radical scavenging activity of different extracts tested.

Sample	SPE	CAE	AF	WPE	GA	D‐O‐R
	IC_50_% mg/g dry extract (mean ± SD)					
	0.046 ± 0.002^e^	0.523 ± 0.001^c^	0.780 ± 0.001^a^	0.613 ± 0.001^b^	0.010 ± 0.0002^f^	0.190 ± 0.006^d^

*Note:* Data represent the mean ± standard deviation of triplicate determinations (*n* = 3). ^a–f^Means in a row with different letters indicate a statistically significant difference (*p* < 0.05); nd: not detected.

Otherwise, the different concentrations of delphinidin‐3‐O‐rutinoside ranged from 0.025 to 0.3 mg/mL presenting a DPPH radical scavenging activity varied from 10.19% to 72.61%. The DPPH radical scavenging activity values for all different samples examined were in the range of 15.07%–79.59%, 22.13%–82.32%, 23.66%–89.58%, and 14.53%–78.49% in Soxhlet, crude water bath extract, crude anthocyanin and separated anthocyanin extracts, respectively, when the concentration varied from 0.01 to 0.076, 0.25 to 1.063, 0.188 to 1.063, 0.188 to 0.813 mg/mL, respectively. Based on various samples examined, the Soxhlet extract demonstrates an important DPPH radical scavenging activity when compared to the other three extracts analyzed. The IC_50_ values of both standards and all peel extracts are given in Table 2. A lower IC_50_ value corresponds to a higher antioxidant activity of plant extract. According to IC_50_ values obtained, the extracts were ranked, in decreasing order, as follows: SPE > CAE > WPE > AF, with IC_50_ values of 0.046, 0.523, 0.613, and 0.78 mg/mL, respectively. As presented in Table 2, the lowest value of IC_50_ in our study was in SPE extract, which is obtained by the Soxhlet method using absolute ethanol as solvent. Although, results of previous research revealed that a mixture of water and ethanol acidified with citric acid is one of the best solvents for phenolic and anthocyanin extraction from the peel of eggplant and other natural sources, which showed the best scavenging activity of DPPH radical (Hosseini et al. 2016). This contradiction could be explained by the presence of lipophilic antioxidants in the SPE extract, as this extract wasn't subject to lipophilic compounds separation. Similarly, the results reported by Deng et al. (2013) indicated that antioxidants in vegetables are either lipophilic or hydrophilic, and lipophilic components contribute mostly to the antioxidant capacities of the vegetables.

Otherwise, these data agreed with the ones obtained from a preceding study, which indicated that ethanol extract can inhibit the formation of free radicals better than water, and increasing the concentration of ethanol increased the antioxidant activity of eggplant (Br Sembring and Chin 2021). This confirms the efficacy of absolute ethanol in extracting phenolic compounds with high antioxidant activity. Thus, the highest values of IC_50_, which correspond to the low value of radical scavenging activity, could be a result of removing these corresponding components from other extracts. All of these results indicated that the extracts of eggplant peel could be used as a good source of natural antioxidants for improving the nutritional value of foods and their preservation, as well as adding them to medical products to replace artificial antioxidants.

The extraction and preparation of phenolic compounds from diverse samples predominantly depend on the characteristics of the sample matrix and the chemical properties of the phenolics themselves, encompassing factors such as molecular structure, polarity, concentration, the number of aromatic rings, and hydroxyl groups. The chemical composition of phenolics within a sample exhibits variability, attributed to the presence of simple and complex polyphenolic compounds, as well as the varying proportions of phenolic acids, flavonoids, anthocyanins, proanthocyanins, and other constituents. Consequently, it becomes challenging to identify a single standardized method for preparing and extracting phenolics in a wide range of plant products (Khoddami et al. 2013).

Concerning the samples extracted using a hot water bath, these three samples, which have high contents of flavonoids, demonstrated a low radical scavenging activity in comparison with SPE sample. This result affirms that reported by Al‐Mamary et al., which showed that the moderate DPPH effect of eggplant extract could be due to multi hydroxy flavonoids, especially OH group in the B‐ring which increased the production of hydroxyl radicals (Ali Al‐Mamary and Moussa 2021). In addition, a previous study stated that quinine derivatives could inhibit the free radical‐induced peroxidation but also play a peroxidation role in the vesicle of dipalmitoyl phosphatidylcholine and this could be due to the electron‐attracting group at the ortho position to hydroxyl group in the phenoxy radical of quinone derivatives which initiation lipid peroxidation (El‐Gammal 2012).

On the other hand, the radical scavenging activity in the AF sample was significantly lower than the one in the CAE sample, which could explain the possibility of a synergistic action among the different classes of polyphenols in the CAE sample, which were not subject to liquid–liquid extraction by solvents of different polarities.

### 3.4 Separation and Identification of Some Phenolic Compounds by HPLC Analysis

The amount of phenolic compounds is an important factor in assessing the quality of various extracts, as it correlates with their resistance to oxidation and antioxidant properties (Moure et al. 2001). To characterize the different phenolic compounds in different extracts obtained and to identify what kinds of polyphenols were found in eggplant peel, HPLC analysis of extracts was performed.

A great variation among the components identified in various extracts of eggplant peel was revealed in Table 3.

**TABLE 3:** Phenolic compounds and their contents in different examined extracts.

Phenolic compounds	AF	WPE
	ppm (Mean ± SD)	
Gallic acid	91.00 ± 8.97	17.50 ± 1.74
Delphinidin 3‐O‐rutinoside	nd	nd
Vanillin	272.00 ± 23.58	8.50 ± 0.77
Quercetin	20.6 ± 2.11	17.1 ± 1.68
Rutin	73.32 ± 7.33	48.89 ± 4.88
Catechin	53.32 ± 5.35	9.87 ± 0.95

*Note:* Data represent the mean ± standard deviation of triplicate determinations (*n* = 6).

Data presented in Table 3 showed that AF and WPE extracts contained five different types of phenolic compounds. The most abundant one for AF extract being vanillin, which comprised about 272 ± 23.58 ppm of the total phenolics, followed by gallic acid 91.00 ± 8.97 ppm. Concerning the other phenolics, rutin, catechin and quercetin are 73.32 ± 7.33, 53.32 ± 5.35 and 20.6 ± 2.11 ppm respectively.

Likewise, the predominant phenolic compound for WPE peels extract was rutin 48.89 ± 4.88 ppm in comparison with the other compounds, followed by gallic acid 17.50 ± 1.74 ppm, quercetin 17.1 ± 1.68 ppm. Unlike the AF extract, catechin and vanillin represent little content for the WPA extract with concentrations 9.87 ± 0.95 and 8.50 ± 0.77 ppm, respectively, while delphinidin 3‐O‐rutinoside was absent in the two extracts. The variation of phenolic compound concentrations between the examined extracts could be due to the effect of different solvent systems and extraction processes used. These results confirm that the high contents of these flavonoid compounds (vanillin, rutin and quercetin) are not attributed to a high antiradical activity of the AF extract.

Otherwise, authors have found that eggplant peel contains high contents of anthocyanins that give different color to various eggplant peels (Azuma et al. 2008). Furthermore, previous results emphasize that various types of delphinidin and nasunin compounds account for large portions of anthocyanins in the peel of eggplant (Sadilova et al. 2006) which confirm that delphinidin 3‐O‐rutinoside is not a predominant anthocyanin in our eggplant peels.

## 4 Conclusions

In view of the results of HPLC analysis, the major polyphenol compounds in separated anthocyanin extract (AF) were vanillin, gallic acid and rutin that accounted for nearly 272 ± 23.58, 91 ± 8.97 and 73.32 ± 7.33 ppm of the total polyphenols, respectively. Likewise, the UV–Vis spectrophotometric analysis revealed that the highest total phenolic content was in crude extract of polypenols obtained by Soxhlet process and the anthocyanins content, as well as condensed tannins were in crude extract of anthocyanin (CAE), (856.71 ± 16.06) mg GA equivalents, (157.29 ± 2.43) mg D‐O‐R, (368.66 ± 2.96) mg C equivalents per g DW, respectively. Otherwise, diverse considerable antioxidant capacities were detected in different extracts examined, which could be important dietary sources of natural antioxidants for the prevention of diseases caused by oxidative stress. With regards to antioxidant activity, the ethanolic extract of Soxhlet method from eggplant peel showed the strongest free radical scavenging activity against DPPH. The Soxhlet crude extract (SPE), which wasn't subject to lipophilic compounds separation, revealed important values of DPPH and TPC tests as compared to other extracts, which suggested that antioxidant components in its lipophilic fraction maybe be scavenging free radicals. Moreover, further experiments should be realized to define which factors are exactly responsible for high antioxidant capacity in the SPE sample.

The findings of this study reflect the importance of dark purple eggplant peel as a valuable source of natural antioxidants useful in medical and food industries, as well as in epidemiological research. This valorization concept allows the conversion of fruit waste into high‐value products with relevant potential applications for human consumption and health. From an economical point of view, utilizing vegetable wastes and by‐products as a natural source of antioxidants can play a significant role in the food industry.

## Author Contributions

**Assia Djouadi:** conceptualization (equal), data curation (equal), formal analysis (equal), investigation (equal), methodology (equal), software (equal), validation (equal), visualization (equal), writing – original draft (equal), writing – review and editing (equal). **Hakan Yılmazer:** project administration (equal), resources (equal), supervision (equal). **Anfal Djouadi:** investigation (equal), methodology (equal). **Bilal Çakır:** data curation (equal).

## Ethics Statement

The authors have nothing to report.

## Conflicts of Interest

The authors declare no conflicts of interest.

## Data Availability

The data that support the findings of this study are available on request from the corresponding author. The data are not publicly available due to privacy or ethical restrictions.
